# The Association of Sleep Duration and Sleep Quality With Depression and Anxiety Among Chinese Commercial Pilots

**DOI:** 10.1155/da/9920975

**Published:** 2024-11-23

**Authors:** Pan Chen, He-Li Sun, Yuan Feng, Qinge Zhang, Tong Leong Si, Zhaohui Su, Teris Cheung, Gabor S. Ungvari, Erliang Zhang, Minzhi Chen, Jie Zhang, Lin Zhang, Bin Ren, Qingqing Jin, Robert D. Smith, Mi Xiang, Yu-Tao Xiang

**Affiliations:** ^1^Unit of Psychiatry, Department of Public Health and Medicinal Administration, Institute of Translational Medicine, Faculty of Health Sciences, University of Macau, Macao SAR, China; ^2^Centre for Cognitive and Brain Sciences, University of Macau, Macao SAR, China; ^3^Beijing Key Laboratory of Mental Disorders, National Clinical Research Center for Mental Disorders and National Center for Mental Disorders, Beijing Anding Hospital, Capital Medical University, Beijing, China; ^4^School of Public Health, Southeast University, Nanjing, China; ^5^School of Nursing, Hong Kong Polytechnic University, Hong Kong SAR, China; ^6^Section of Psychiatry, University of Notre Dame Australia, Fremantle, Australia; ^7^Division of Psychiatry, School of Medicine, University of Western Australia, Perth, Australia; ^8^School of Public Health, Shanghai Jiao Tong University, Shanghai, China; ^9^CAAC East China Aviation Personnel Medical Appraisal Center, Shanghai 200336, China; ^10^Hainan Branch, Shanghai Children's Medical Center, School of Medicine, Sanya and School of Public Health, Shanghai Jiao Tong University, Shanghai, China

**Keywords:** anxiety, commercial pilots, depression, sleep duration, sleep quality

## Abstract

**Background:** Sleep problems are known as risk factors for depression and anxiety, but research on this subject with commercial pilots is limited. This study aimed to explore the effects of sleep problems on depressive and anxiety symptoms among Chinese commercial pilots.

**Methods:** Adults who participated in the baseline assessment of the Civil Aviation Health Cohort of China between December 2022 and March 2023 formed the study sample. Depressive and anxiety symptoms and sleep quality were assessed using standardized scales. Sleep duration was measured with standardized questions. Logistic regression and restricted cubic splines (RCSs) were used to analyze the association between sleep problems and depression/anxiety symptoms.

**Results:** A total of 7055 pilots were included in this study. The overall prevalence of depression and anxiety among pilots was 23.3% (*n* = 1642; 95% confidence interval [CI] = 22.3%–24.3%) and 17.0% (*n* = 1196; 95% CI = 16.1–17.8%), respectively. Logistic regression analyses revealed that short sleep duration (<7 h) was significantly associated with a higher risk of depression (odds ratio [OR] = 2.491; *p*  < 0.001) and anxiety (OR = 2.555; *p*  < 0.001), while poor sleep quality was also associated with a higher risk of depression (OR = 7.297; *p*  < 0.001) and anxiety (OR = 7.469; *p*  < 0.001). After adjusting for confounders, there was an inverse, J-shaped nonlinear relationship between sleep duration and both depression (inflection point: 7.64 h) and anxiety (inflection point: 7.48 h). Similarly, a J-shaped nonlinear relationship was found between sleep quality and depression/anxiety with an inflection point of Pittsburgh Sleep Quality Index (PSQI) = 4 points for both. The major limitation of the study was that causal relationships between variables could not be inferred due to the cross-sectional study design.

**Conclusion:** This study found that depression and anxiety were common among Chinese commercial pilots. Insufficient length and poor quality of sleep were associated with an increased risk of depression and anxiety. Implementing targeted strategies to improve sleep patterns is crucial for reducing the risk of depression and anxiety in this population.

## 1. Introduction

The mental health of special occupational populations has gained increased attention, particularly among those closely associated with public safety, such as pilots, healthcare workers, police officers, and bus drivers. Air crashes caused by human factors have alerted the public about the need to pay attention to the mental health of pilots. For instance, in the case of “Germanwings Flight 4U 9525,” all 150 people on board died tragically due to deliberate maneuvering by the first officer, who had a relapse of depression [1]. Another one, in the case of “JetBlue Flight 191,” the first officer timely recognized the suspicious behavior of the captain who appeared to be having a panic attack, thus ensuring the safety of the passengers [2, 3]. Depressive symptoms and anxiety symptoms were commonly found in pilots [4, 5]. A review concluded that the global prevalence of depression among pilots ranged from 1.9% to 12.6% [6]. A Chinese survey reported that 26.2% of pilots experienced anxiety symptoms [5]. High workload, extended duty hours [7], sudden air crash events (i.e., China Eastern Airlines Flight 5735) [8], and the emergent public health issues (i.e., the COVID-19 pandemic) in recent years as other stressors may have contributed to elevated risk of depression and anxiety [9, 10], which could lead to a number of adverse health outcomes and poor quality of life (QOL) [2]. However, to date, only a few studies have investigated the prevalence of depression and anxiety among commercial pilots.

Sleep problems have also been a great concern among pilots, which are not only manifested as symptoms but also acted as risk factors for mental health problems. Both the quantity and quality of sleep were crucial for maintaining normal or productive work and personal lives [11], especially for people who have heavy workloads and shift work [12]. Previous surveys found high levels of fatigue, sleep problems, and mental health issues among both short- and long-haul pilots [13, 14]. A previous study on pilots showed that up to half of them were at risk of developing insomnia [15], which would jeopardize aviation safety. Abnormal sleep duration (i.e., either less or more sleep) could increase the risk of accidental injury and death [16, 17] and affect QOL and cognitive function [18, 19]. Moreover, poor sleep quality is associated with increased risk of various adverse physical and psychological health outcomes such as obesity, cardiovascular diseases, depression, anxiety, and even suicidality [20–22].

Sleep problems, depression, and anxiety could interact with each other [23]; therefore, understanding their relationships was crucial for developing preventive or treatment strategies and allocating health resources. To date, studies have examined the relationship between sleep problems and depression and anxiety across various populations, such as children and adolescents, older adults, university students, and the general population [20, 23–27]. Previous studies reported inconsistent findings, which may be partly due to different sampling methods and differing sociocultural contexts. Several studies on the linear relationship between sleep problems and levels of depression and anxiety have reported a negative association between shorter sleep duration and depression and anxiety [28, 29]. However, recent evidence has revealed a nonlinear relationship between sleep disturbances and depression and anxiety [24, 26, 30, 31]. For instance, a study conducted in older Chinese adults found a U-shaped relationship between nighttime sleep duration and depression, as well as a J-shaped relationship between sleep quality and depression [32]. These findings suggest that both inadequate and excessive sleep duration, along with poor sleep quality, elevate the risk of developing depression. Conversely, some studies have demonstrated a reverse J-shaped, nonlinear relationship between sleep duration and depression, indicating that longer sleep duration is associated with a decreased risk of depressive symptoms [31, 33]. However, larger-scale studies specifically on pilots are lacking.

The relationship between sleep problems and depression and anxiety among Chinese pilots is still unclear. Thus, this study aimed to (1) investigate the prevalence of depression and anxiety among Chinese commercial pilots and (2) explore the specific relationship of sleep duration and quality with depression and anxiety in the same population. Given previous findings [4, 34], we hypothesized that depression and anxiety among Chinese commercial pilots would be common and there would be nonlinear relationships between sleep problems and depression and anxiety.

## 2. Methods

### 2.1. Study Design and Study Population

This study was based on the baseline assessment of the Civil Aviation Health Cohort of China (CAHCC), a national survey of the physical and mental health of crew members. The survey was conducted online between December 2022 and March 2023, involving 10 Chinese commercial airlines. The inclusion criteria were as follows: (1) age 18 years or above; (2) employed as pilots in the participating commercial airlines during the study period; and (3) voluntary participation in the study. There were no specific exclusion criteria. The study was approved by the Ethics Committee of Civil Aviation Shanghai Hospital (No. 2021-7). All participants provided electronic written informed consent.

### 2.2. Measures

#### 2.2.1. General Characteristics

Sociodemographic and clinical characteristics of the participants were collected, including age, gender, education level, living status, perceived income level, employment as pilots (years), global QOL, and COVID-19-related information (i.e., history of COVID-19 infection, being close contact or suspected close contact, and having quarantined during the COVID-19 pandemic). The global QOL was measured by summing the first two items of the self-reported World Health Organization Quality of Life Brief Version (WHOQOL-BREF) [35–37]. A higher total score indicated a higher QOL.

#### 2.2.2. Sleep Duration and Quality

Following previous research [38], sleep duration was measured using three standardized questions for three conditions: “During the past month, on days with early morning flights, how many hours did you actually sleep per day?”; “During the past month, on days with late evening flights, how many hours did you actually sleep per day?”; and “During the past month, on days with rest days, how many hours did you actually sleep per day? (Note: Actual sleep duration may be shorter than the number of hours you spend in bed).” The sleep duration (hours) for each individual was defined as the average daily sleep duration across three situations: on days with early morning flights, on days with late evening flights, and on days with rest days. According to the recommendation from the National Sleep Foundation [39, 40], 7–9 h were considered “normal sleep duration,” while less than 7 h and more than 9 h were considered “short” and “long” sleep durations, respectively.

Sleep quality was assessed using the validated Chinese version of the self-reported Pittsburgh Sleep Quality Index (PSQI) [41–43]. The PSQI consists of 19 items, covering seven components: subjective sleep quality, sleep latency, sleep duration, habitual sleep efficiency, sleep disturbances, use of sleeping medications, and daytime dysfunction. Each component was scored from 0 to 3; thus, the total score ranged from 0 to 21, with higher scores indicating poorer sleep quality. Following previous studies [44, 45], the total score of PSQI greater than 5 was considered indicative of “poor sleep quality.”

#### 2.2.3. Depressive and Anxiety Symptoms

Depression was assessed using the validated Chinese version of the self-reported Patient Health Questionnaire 9-item (PHQ-9) [46, 47]. Each item was rated on a 4-point Likert scale from 0 (“not at all”) to 3 (“nearly every day”). The total scores of the PHQ-9 ranged from 0 to 27. A cutoff value of 5 was considered as “having a depressive syndrome (depression hereafter)” [48, 49].

Anxiety was assessed using the validated Chinese version of the self-reported General Anxiety Disorder scale 7-item (GAD-7) [50, 51]. Each item was rated on a 4-point Likert scale from “0” (not at all) to “3” (nearly every day). The total scores of the GAD-7 (0 to 21) ranged from 0 to 21. A cutoff value of 5 was considered as “having an anxiety syndrome (anxiety hereafter)” [49, 51] .

### 2.3. Data Analysis

#### 2.3.1. Univariate and Multivariate Analyses

All analyses were performed using R version 4.3.2 [52]. The normality of distribution for continuous variables was tested using the Kolmogorov–Smirnov test. To compare the demographic and clinical characteristics between the depression and nondepression groups and between the anxiety and nonanxiety groups, independent sample *t*-tests, Mann–Whitney *U* tests, and Chi-square tests were employed, as appropriate.

Binary logistic regression analyses with the “enter” method were performed to examine the independent association of sleep variables (sleep duration/quality as independent variables) with depression or anxiety (dependent variables) after adjusting for confounders. Variables with significant group differences in univariate analyses (*p*  < 0.05) were considered as potential confounders of having depression or anxiety. Adjusted odds ratios (ORs) and 95% confidence intervals (CIs) were calculated to estimate the strength of the associations. Statistical significance was set at *p*  < 0.05 for all analyses (two-tailed).

Restricted cubic spline (RCS) is a commonly used approach for describing the dose–response relationship between continuous exposure and outcomes when a nonlinear correlation is anticipated [53]. RCS curves were fitted with four knots to further explore the potential nonlinear relationship between sleep variables (sleep duration/quality) and depression and anxiety. Four knots were positioned at the 5th, 35th, 65th, and 95th percentiles of the sleep duration (i.e., 6.0, 7.3, 8.0, and 9.0 h) and the PSQI score (0, 3, 5, and 10 points) [54, 55]. A *p* value of <0.05 indicated a nonlinear relationship.

## 3. Results

### 3.1. Sociodemographic Characteristics

Of the 8640 pilots invited to participate in this study, 7918 agreed to participate and completed the CAHCC survey assessment, giving a participation rate of 91.6%. Eventually, 7055 pilots met the study entry criteria and were included in the analysis. Participants' demographic and clinical characteristics are shown in Table 1. The mean age of the sample was 34.1 (standard deviation [SD] = 6.94) years. Most pilots (*n* = 4958; 70.3%) had a history of COVID-19 infection, and over one-third (*n* = 2664; 37.8%) were quarantined during the COVID-19 pandemic. The sleep duration ranged from 4 to 13 h (mean = 7.4, SD = 0.92), and the mean PSQI score was 4.5 (SD = 2.87).

### 3.2. Prevalence of Depression and Anxiety

The overall prevalence of depression (PHQ-9 total score ≥ 5) and anxiety (GAD-7 total score ≥ 5) among pilots was 23.3% (*n* = 1642; 95% CI = 22.3%–24.3%) and 17.0% (*n* = 1196; 95% CI = 16.1%–17.8%), respectively. The total score of the PHQ-9 and GAD-7 in the whole sample was 2.69 (SD = 4.036) and 1.78 (SD = 3.245), respectively.

### 3.3. Associations Between Sleep Duration, Sleep Quality, and Depression and Anxiety

In univariate analyses (Table 1), participants with depression were more likely to have a short sleep duration (<7 h; 35.4% vs. 15.2%; *p*  < 0.001) and poor sleep quality (PSQI >5; 69.4% vs. 22.1%; *p*  < 0.001) compared to the nondepression group. Participants with anxiety were more likely to have a short sleep duration (<7 h; 37.5% vs. 17.2%; *p*  < 0.001) and poor sleep quality (PSQI > 5; 73.2% vs. 24.9%; *p*  < 0.001) compared to the nonanxiety group. Participants with depression (*p*  < 0.001) or anxiety (*p*  < 0.001) were more likely to report a lower QOL.

**Figure 1:**
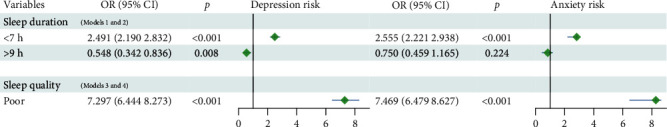
Logistic regression models between sleep duration, sleep quality, and depressive and anxiety symptoms in pilots. CI, confidence interval; OR, odds ratio.

As shown in Table 2 and Figure 1, after adjusting for confounders, logistic regression analyses indicated that compared to the normal sleep duration group (7–9 h), participants with short sleep duration (<7 h) had a significantly higher risk of depression (OR = 2.491; *p*  < 0.001) and anxiety (OR = 2.555; *p*  < 0.001). Compared to the normal sleep duration group (7–9 h), however, only those with a long sleep duration (>9 h) had a significantly lower risk of depression (OR = 0.548; *p*=0.008), while no significant association between long sleep duration and risk for anxiety was found (OR = 0.750;*p*=0.223). In addition, participants with poor sleep quality (PSQI > 5) had seven times higher risk of depression (OR = 7.297; *p*  < 0.001) and anxiety (OR = 7.469; *p*  < 0.001) compared to those with good sleep quality.

### 3.4. J-Shaped Nonlinear Relationship Between Sleep Duration/Quality and Depression and Anxiety

**Figure 2:**
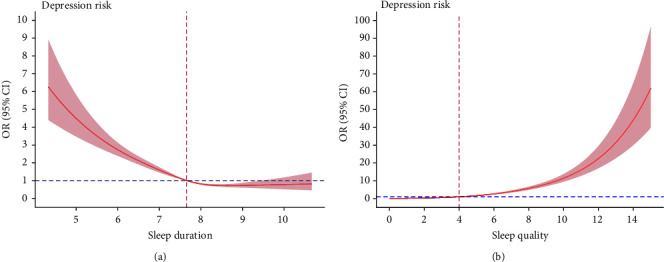
Nonlinear association of sleep duration (a), sleep quality (b), and depression. CI, confidence interval; OR, odds ratio.

**Figure 3:**
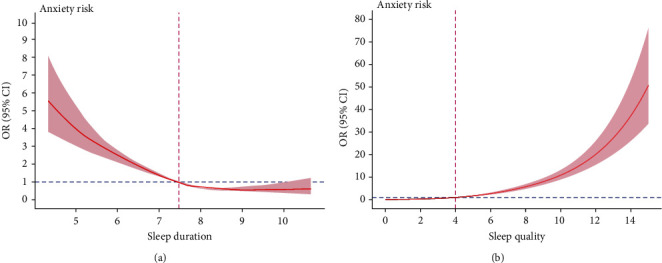
Nonlinear association of sleep duration (a), sleep quality (b), and anxiety. CI, confidence interval; OR, odds ratio.

As shown in Figures 2 and 3, after adjusting for confounders, there was an inverse J-shaped nonlinear relationship between sleep duration and depression with an inflection point (OR = 1) of 7.64 h. Similarly, an inverse J-shaped nonlinear relationship between sleep duration and anxiety was found with an inflection point of 7.48 h. In addition, J-shaped nonlinear relationship between sleep quality and depression and anxiety was also found, with an inflection point of PSQI = 4 points (OR = 1) for both. The *p* values for all nonlinearity values were less than 0.001.

## 4. Discussion

To the best of our knowledge, this was the first large-scale study that investigated the prevalence of depression and anxiety among Chinese commercial pilots and explored the nonlinear relationship of sleep duration and sleep quality with depression and anxiety in this population. The main findings of this study were that depression and anxiety were common among Chinese commercial pilots. Commercial pilots with depression and anxiety were also more likely to have lower sleep duration and poorer sleep quality compared to those without these sleep disturbances.

### 4.1. High Prevalence of Depression and Anxiety Among Pilots

The prevalence of depression (PHQ-9 total score ≥ 5) and anxiety (GAD-7 total score ≥ 5) among pilots was 23.3% (95% CI = 22.3%–24.3%) and 17.0% (95% CI = 16.1%–17.8%), respectively. These rates indicated an elevated or comparable risk compared to the Chinese general population, with rates of 17.0% for depression and 18.0% for anxiety during the COVID-19 pandemic [56]. When compared to the findings in the Chinese general population prior to the COVID-19 pandemic (depression: 17.9%; anxiety: 11.0%), pilots appeared to be at higher risk of both depression and anxiety [57, 58].

These findings differed from those reported in previous studies on pilots. For example, an online survey conducted in over 50 countries found that 12.6% of pilots suffered from moderate or severe depression (PHQ-9 ≥ 10) [59]. Another study in China found that the prevalence of anxiety among pilots was 26.2%, as measured with the Zung Self-Assessment Scale for Anxiety (SAS) with a cutoff value of 50 [5]. Similar surveys were conducted in Australia (17.2% with depression and 7.8% with anxiety) and the European Aviation Safety Agency (EASA) (18.0% with depression and 8.5% with anxiety), measured using the PHQ-8 (a score of ≥ 10 indicating depression) and GAD-7 (a score of ≥ 10 indicating anxiety; the GAD-7 total score was 3.94 ± 3.63 in Australia and 3.76 ± 3.76 in EASA) [14]. These inconsistent findings in both the prevalence and severity of depression and anxiety may be related to the differences in measurement criteria, sample sizes, and geographic regions with different sociocultural backgrounds [60]. Wu et al. [58] found that pilots from countries dominated by Western cultural traditions tended to have a lower prevalence of depression. The high prevalence of depression and anxiety in pilots can be attributed to multiple reasons, such as occupational stress (i.e., high workload and shift work) [7], unhealthy lifestyle [61], low income level [5], and adverse working or life experiences (e.g., substance abuse and verbal or sexual abuse) [6]. For example, there is evidence that pilots with longer hours of duty were more likely to report feeling depressed or anxious [4]. Another study found that Chinese airline pilots were paid much less than their foreign counterparts. This imbalance between high workload and lower income level could increase the level of negative emotions and occupational stress leading to anxiety [5].

### 4.2. J-Shaped Relationship Between Sleep Duration and Depression and Anxiety

Consistent with a previous finding in adolescents [62], this study revealed the nonlinear relationships between sleep duration and depression and anxiety among Chinese pilots after adjusting for confounders. The RCS results further indicated that shorter sleep duration was the risk factor for depression (<7.64 h) and anxiety (<7.48 h). Insufficient sleep was a recognized risk factor for both physical and mental conditions such as increased incidence of cardiovascular diseases, obesity and related metabolic syndrome, cancer, cognitive deficit, mood dysregulation, irritability, depression, anxiety, and even suicide [63–65]. For airline pilots, the leading consequence of insufficient sleep could be fatigue, decreasing concentration or alertness during duty, which may trigger and further elevate the risk of depression and anxiety [6, 30, 66]. Additionally, from a biological perspective, there is compelling evidence that the association between sleep and mental problems, such as depression and anxiety, was mediated by inflammation [67]. Sleep deprivation can lead to proinflammatory state [67, 68], such as increased sensitivity of inflammatory cytokines and the change in the level of brain-derived neurotrophic factor (BDNF) [69, 70]. Inflammation is a predictor of depression and anxiety [71–73]. Thus, sleep disturbances have been identified as a significant vulnerability factor to increase the risk of mental disturbances in the presence of inflammation [40, 74].

The present study also found that long sleep duration (>9 h) remained a protective factor for depression in pilots, as indicated by the logistic regression analysis. This is different from previous findings that found that excessive sleep increased the risk of mental health problems [18, 62, 75]. Previous studies proposed several potential mechanisms for the findings that long sleep duration could increase the risk of mental health disturbances [24, 62]. For example, excessive sleep may be associated with increased sleep fragmentation and reduced physical activities, leading to lower energy and vitality as well as mood dysregulation [76–78]. Furthermore, excessive sleep may be a consequence of stress or stressful events, while stress-coping deficits may be a driver of the relationship between sleep and depression [79]. The inconsistency between the current and previous findings may be related to the different distributions of sleep duration across the samples; in this study, the proportion of long sleepers was low (2.9%), whereas in the study of Chinese older adults aged ≥65 years, it was 8.2% [75]. Other reasons included different confounders adjusted for [32] as well as inconsistent definitions of long sleep duration [32, 62] and the age of participants [24].

### 4.3. J-Shaped Relationship Between Sleep Quality and Depression and Anxiety

Similar nonlinear relationships were also observed between sleep quality and depression and anxiety in this study. A PSQI score of >4 was identified as a risk factor for both depression and anxiety. Our findings are consistent with previous studies, indicating that individuals with poor sleep quality were more likely to be depressed or anxious than those with good sleep quality [21, 80, 81]. Sleep quality refers to individuals' satisfaction with all aspects of their sleep, which is affected by multiple factors, including physiological (e.g., age, gender, and circadian rhythm), psychological (e.g., stress, anxiety, and depression), and environmental factors (e.g., noise) [22]. Previous research on pilots found that distressing shifts were associated with difficulty of falling asleep and that low social support and high workloads were associated with subjective poor sleep quality [82]. Pilots often fly early and late shifts, which could frequently change the sleep rhythms. Circadian rest–activity rhythm disturbances are associated with higher risk of mental health disturbances such as depression and anxiety [83] and could even lead to suicide and self-injury [84]. Additionally, there is an overlap between the neural mechanisms of emotional regulation and sleep regulation; thus, impaired sleep quality could disrupt emotional regulation and increases the risk of depressive and anxiety symptoms [85].

### 4.4. Strengths and Limitations

The strengths of this study included its large sample size and the multicenter study design. In addition, the high participation rate (91.6%) and the use of standardized outcome measures enabled the direct comparison of the results with those of other populations. However, several limitations need to be noted. First, causal relationships between variables could not be inferred due to the cross-sectional study design. Second, the use of self-reported measurements may result in recall bias. Third, although a few confounders were adjusted for to explore the independent relationships between sleep and depression and anxiety, there was still a risk to residual confounding, such as personality traits [86], physical comorbidities [87], and adverse childhood experiences [88].

## 5. Conclusions

Depression and anxiety were common among Chinese commercial pilots and were significantly associated with sleep duration and sleep quality in this study. Insufficient sleep, along with poor sleep quality, was linked to an increased risk of depression and anxiety. These findings underscore the importance of managing airline pilots' sleep disturbances and promote their psychological well-being. This study has practical implications. Both sleep length and quality play important roles in developing mental health disturbances, especially depression and anxiety. Thus, implementing targeted interventional strategies to improve sleep patterns is crucial for reducing the risk of mental health problems and mitigating the potential negative impact on the psychological health of this population. The interventional strategies include arranging more convenient duty schedules for pilots to ensure adequate rest breaks, thereby promoting mental and physical recovery after stressful workdays; providing training on proper sleep hygiene practices (e.g., regular sleep schedule, suitable physical activities, and conducive sleep environment); increasing support for preventive measures (e.g., regular health checkups); and implementing mobile health (mHealth) intervention via mobile technologies and Internet-based cognitive behavior therapy [59, 89–91].

## Figures and Tables

**Table 1:** Demographic and clinical characteristics with respect to depressive and anxiety symptoms among pilots.

Variables	Total	Depressive symptoms (PHQ-9 ≥ 5)		Univariable analysis		Anxiety symptoms (GAD-7 ≥ 5)		Univariable analysis	
		Yes (*n* = 1642)	No (*n* = 5413)			Yes (*n* = 1196)	No (*n* = 5859)		
	*n* (%)	*n* (%)	*n* (%)	*χ*2	*p*	*n* (%)	*n* (%)	*χ*2	*p*
Male	6978 (98.9)	1631 (99.3)	5347 (98.8)	3.032	0.082	1191 (99.6)	5787 (98.8)	5.321	**0.021**
College and above	6914 (98.0)	1619 (98.6)	5295 (97.8)	3.518	0.061	1182 (98.8)	5732 (97.8)	4.545	**0.033**
Living with others	5617 (79.6)	1265 (77.0)	4352 (80.4)	8.553	**0.003**	923 (77.2)	4694 (80.1)	5.118	**0.024**
Satisfied with income level	3750 (53.2)	612 (37.3)	3138 (58.0)	215.960	**<0.001**	416 (34.8)	3334 (56.9)	194.310	**<0.001**
History of COVID-19 infection	4958 (70.3)	1220 (74.3)	3738 (69.1)	16.333	**<0.001**	870 (72.7)	4088 (69.8)	4.052	**0.044**
Identified or suspected as a close contact during the COVID-19 pandemic	942 (13.4)	273 (16.6)	669 (12.4)	19.459	**<0.001**	212 (17.7)	730 (12.5)	23.357	**<0.001**
Being quarantined during the COVID-19 pandemic	2,664 (37.8)	713 (43.4)	1951 (36.0)	28.881	**<0.001**	528 (44.1)	2136 (36.5)	24.669	**<0.001**
Sleep duration									
<7 h	1457 (20.7)	581 (35.4)	876 (16.2)	290.410*⁣*^*∗*^	**<0.001**	449 (37.5)	1008 (17.2)	252.100*⁣*^*∗*^	**<0.001**
7–9 h	5394 (76.5)	1038 (63.2)	4356 (80.5)	—	**—**	726 (60.7)	4668 (79.7)	—	**—**
>9 h	204 (2.9)	23 (1.4)	181 (3.3)	—	**—**	21 (1.8)	183 (3.1)	—	**—**
Poor sleep quality (PSQI > 5)	2,334 (33.1)	1,140 (69.4)	1,194 (22.1)	1274.800	**<0.001**	875 (73.2)	1459 (24.9)	1042.700	**<0.001**

	**Mean (SD)**	**Mean (SD)**	**Mean (SD)**	***Z***	***p***	**Mean (SD)**	**Mean (SD)**	***t/Z***	***p***

Age	34.1 (6.94)	34.0 (6.81)	34.1 (6.97)	−0.360	0.719	33.9 (6.63)	34.1 (7.00)	−0.185	0.854
BMI	23.8 (2.33)	23.9 (2.45)	23.7 (2.29)	−3.515	**<0.001**	23.9 (2.40)	23.7 (2.32)	−2.067	**0.039**
Work years	9.2 (7.53)	9.1 (7.35)	9.2 (7.58)	−0.653	0.514	9.2 (7.10)	9.1 (7.61)	−1.593	0.111
QOL	7.1 (1.45)	6.1 (1.26)	7.4 (1.36)	−32.362	**<0.001**	6.0 (1.32)	7.3 (1.37)	−28.985	**<0.001**
Sleep duration (h)	7.4 (0.92)	7.1 (0.98)	7.6 (0.87)	−17.638	**<0.001**	7.0 (0.98)	7.5 (0.88)	−16.794	**<0.001**
Sleep quality (PSQI total score)	4.5 (2.87)	7.1 (2.85)	3.8 (2.39)	—	**<0.001**	7.3 (2.92)	4.0 (2.50)	−34.375	**<0.001**

*Note*: Bold values: <0.05.

Abbreviations: BMI, body mass index; GAD-7, General Anxiety Disorder 7-item; PHQ-9, Patient Health Questionnaire 9-item; PSQI, Pittsburgh Sleep Quality Index; SD, standard deviation; QOL, quality of life.

*⁣* ^*∗*^degree of freedom = 2, others = 1.

**Table 2:** Results of logistic regression analyses between sleep problems and depressive/anxiety symptoms in pilots.

Variables	Depressive symptoms^a^			Anxiety symptoms^b^		
	*p*	OR	95% CI	*p*	OR	95% CI
Sleep duration						
<7 h (short)	**<0.001**	2.491	2.190–2.832	**<0.001**	2.555	2.221–2.938
7–9 h (normal, reference group)	—	—	—	—	—	—
>9 h (long)	**0.008**	0.548	0.342–0.836	0.223	0.750	0.459–1.165
Sleep quality						
Good (PSQI ≤ 5, reference group)	—	—	—	—	—	—
Poor (PSQI > 5)	**<0.001**	7.297	6.444–8.273	<0.001	7.469	6.479–8.627

*Note*: Bold values signifies *p* < 0.05.

Abbreviations: BMI, body mass index; CI, confidence interval; OR, odds ratio; PSQI, Pittsburgh Sleep Quality Index.

^a^Adjusted for BMI, living status, income level, history of COVID-19 infection, identified as a close contact or suspected close contact, being quarantined.

^b^Adjusted for gender, education, living status, BMI, income level, history of COVID-19 infection, identified as a close contact or suspected close contact, being quarantined.

## Data Availability

The datasets presented in this article are not readily available because the Ethics Committee of Civil Aviation Shanghai Hospital (No. 2021-7) that approved the study prohibits the authors from making publicly available the research dataset of clinical studies. Requests to access the datasets should be directed to xyutly@gmail.com.
